# Impact of Inorganic Ions and Organic Matter on the Removal of Trace Organic Contaminants by Combined Direct Contact Membrane Distillation–UV Photolysis

**DOI:** 10.3390/membranes10120428

**Published:** 2020-12-15

**Authors:** Arbab Tufail, William E. Price, Faisal I. Hai

**Affiliations:** 1 Strategic Water Infrastructure Laboratory, School of Civil, Mining and Environmental Engineering, University of Wollongong, Wollongong, NSW 2522, Australia; at742@uowmail.edu.au; 2 Strategic Water Infrastructure Laboratory, School of Chemistry and Molecular Bioscience, University of Wollongong, Wollongong, NSW 2522, Australia; wprice@uow.edu.au

**Keywords:** photodegradation, membrane distillation, halide ions, nitrate ions, carbonate ions, humic acid, trace organic contaminants

## Abstract

This study investigated the degradation of five trace organic contaminants (TrOCs) by integrated direct contact membrane distillation (DCMD) and UV photolysis. Specifically, the influence of inorganic ions including halide, nitrate, and carbonate on the performance of the DCMD–UV process was evaluated. TrOC degradation improved in the presence of different concentrations (1–100 mM) of fluoride ion and chloride ion (1 mM). With a few exceptions, a major negative impact of iodide ion was observed on the removal of the investigated TrOCs. Of particular interest, nitrate ion significantly improved TrOC degradation, while bicarbonate ion exerted variable influence—from promoting to inhibiting impact—on TrOC degradation. The performance of DCMD–UV photolysis was also studied for TrOC degradation in the presence of natural organic matter, humic acid. Results indicated that at a concentration of 1 mg/L, humic acid improved the degradation of the phenolic contaminants (bisphenol A and oxybenzone) while it inhibited the degradation of the non-phenolic contaminants (sulfamethoxazole, carbamazepine, and diclofenac). Overall, our study reports the varying impact of different inorganic and organic ions present in natural water on the degradation of TrOCs by integrated DCMD–UV photolysis: the nature and extent of the impact of the ions depend on the type of TrOCs and the concentration of the interfering ions.

## 1. Introduction

Trace organic contaminants (TrOCs)—namely pharmaceuticals and personal care products, pesticides, surfactants, and industrial reagents—occur at the level of nanogram to microgram per litre in wastewater and polluted waterbodies [1,2,3]. The presence of these contaminants even in trace concentrations in the environment raises concern because of their harmful effects on human beings and aquatic lives [4]. A systematic analysis of the available studies shows that conventional wastewater treatments are not capable of effective elimination of persistent TrOCs. This results in their occurrence in the surface water and groundwater [5,6,7]. Therefore, an effective treatment process is essential for TrOC removal.

Membrane distillation involves moderate-temperature distillation compared to conventional distillation processes (e.g., steam distillation). In this process, water is transported in vapour form from the feed side to distillate through porous hydrophobic membranes. Among the available configurations, direct contact membrane distillation (DCMD) is a promising technology [8,9,10]. This is a high-retention membrane process that involves low fouling potential compared to pressure-driven membrane processes and can be less energy-intensive when low-grade waste heat is used [11,12]. A few recent studies have reported the removal of TrOCs by this process [13,14,15]. For TrOC removal, a temperature difference of 15–20 °C is created to drive the transport of water vapour from feed to distillate. Non-volatile TrOCs can be effectively removed by the DCMD system as mass transport occurs in the vapour phase [16,17]. The TrOCs effectively retained by DCMD (i.e., the membrane concentrate) require additional treatment before disposal to the environment [18]. We propose that the DCMD system can be integrated with UV photolysis, thereby devising the DCMD–UV photolysis process, where the DCMD membrane can retain the contaminants and UV photolysis can continuously degrade them.

More commonly applied in water disinfection, UV photolysis can also be applied for the degradation of TrOCs. The literature shows that contaminants with photolabile moieties (e.g., -Cl, -OH) absorb photon energy from UV light and undergo oxidation [19,20,21]. There is limited information on overall TrOC removal by combined DCMD–UV photolysis. Mozia et al. [22] investigated diclofenac removal by combined DCMD and UV photolysis and revealed its complete elimination within 4 h. Another study reported the removal of TrOCs by DCMD preceding UV photolysis and showed 27–88% removal depending on the TrOC [18]. 

DCMD can effectively retain the non-volatile TrOCs and UV photolysis can degrade them with TrOC-specific efficiency. However, the presence of inorganic ions—namely halide ions, nitrate ions, and bicarbonate ions—in water can influence TrOC removal efficiency [20,23,24]. For instance, Li et al. [25] demonstrated the effect of bromide and chloride ions on the photodegradation of three antibiotics (sulfamethoxazole, sulfamethazine, and sulphapyridine) and reported improved rate constants for sulfamethazine and sulphapyridine in the presence of chloride ion while the rate constants decreased for sulfamethoxazole. In that study, the rate constant for all the TrOCs decreased in the presence of bromide ion. Yang et al. [26] reported the inhibitory effect of bicarbonate ion on sulfamethoxazole photodegradation. Chloride and nitrate ions also inhibited the photodegradation of diclofenac, while bicarbonate ion enhanced diclofenac degradation [26]. The efficiency of the DCMD–UV treatment may be also affected in the presence of natural organic matter such as humic acid in water. The presence of humic acid gives water a yellowish-brown colour which can reduce UV light penetration. Humic acid also acts as an OH radical scavenger. However, the literature illustrates contrasting results regarding the effect of humic acid on the UV photolysis of TrOCs. For instance, the degradation rate constant of 17α-ethinylestradiol and 17β-estradiol greatly improved in the presence of humic acid [27,28] while naproxen degradation was inhibited [29]. Overall, information on the impact of co-occurring ions in water on TrOC degradation by DCMD–UV is very limited.

This study investigated TrOC retention by DCMD and their degradation by combined DCMD–UV photolysis using five selected TrOCs including bisphenol A, oxybenzone, diclofenac, carbamazepine, and sulfamethoxazole, which have diverse physicochemical properties and are commonly detected in wastewater and polluted waterbodies. Because the presence of organic and inorganic ions in water may significantly influence TrOC degradation, the effect of inorganic ions such as halide, nitrate, and bicarbonate ions as well as natural organic matter (i.e., humic acid) on DCMD–UV photolysis was systematically studied. This is the first study to elucidate the effect of different interfering ions on TrOC removal from water by the integrated DCMD–UV system.

## 2. Materials and Methods

### 2.1. Materials

In this study, five TrOCs including bisphenol A, oxybenzone, diclofenac, carbamazepine, and sulfamethoxazole were selected in view of their common detection in wastewater-affected natural waterbodies [1]. Table 1 illustrates the main physicochemical characteristics of these TrOCs, namely contaminant structures, hydrophobicity, and dissociation constants. All tested TrOCs were bought from Sigma Aldrich (Castle Hill, Australia) and had greater than 98% purity. Inorganic salts (i.e., sodium fluoride, sodium chloride, sodium bromide, sodium iodide, sodium nitrate, and sodium bicarbonate), humic acid, methanol, and HPLC-grade acetonitrile were also sourced from the same supplier. Ultrapure Milli-Q water (Millipore S.A.S, Molsheim, France) was used in all experiments and each experiment was conducted in duplicate. According to the supplier, Milli-Q water has a resistivity of 18.2 MΩ·cm and a total organic carbon (TOC) of less than 5 ppb.

### 2.2. Sample Preparation

A stock solution of the selected TrOCs was prepared by adding the TrOCs each at a concentration of 2 g/L to pure methanol. The stock solution was stored in the dark at −18 °C and used within one month. A working solution was freshly prepared by diluting the stock solution in Milli-Q water to obtain 1 mg/L concentration of the TrOCs. A calibration curve of each TrOC was established in the range 0.1–1 mg/L to quantify TrOC concentration in samples by HPLC. Stock solutions (1 M) were prepared for each inorganic salt. Humic acid stock solution was at a concentration 1 g/L. These solutions were further diluted to obtain working solutions of the salts (1 5, 10, and 100 mM) and humic acid (1, 5, and 10 mg/L).

### 2.3. DCMD and UV Setup and Operation Protocol

**Figure 1:**
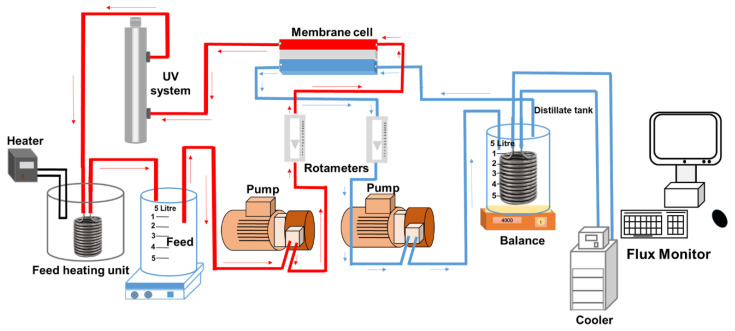
Experimental setup for integrated direct contact membrane distillation–UV photolysis treatment.

A lab-scale membrane distillation rig comprising a direct contact membrane cell and a reactor made of glass, as shown in Figure 1, was used to conduct experiments. The liquid in the glass reactor was used as feed for the DCMD module. The feed tank (working volume of 5 L) was placed in a temperature controlled (30 ± 1 °C) water bath. The water bath was equipped with a heating unit (Julabo, Seelbach, Germany) to keep the feed temperature at 30 ± 1 °C. Temperature for distillate was set up at 10 ± 1 °C using a chiller (SC100-A10, Thermo Scientific, Vernon Hills, IL, USA). The setup was designed and operated following a previously published protocol [16]. The initial feed volume was 1.5 L and the nominal concentration of each TrOC in the feed solution was 1 mg/L. The impact of different concentrations of each ion (1, 5, 10, and 100 mM) and humic acid (1, 5, and 10 mg/L) was studied in separate runs. In each run, the DCMD–UV setup was run for 60 min. The DCMD system was run in recirculation mode and flow rate was kept at 1 L/min for both feed and permeate. The permeate flux was recorded every 5 min and the DCMD system was initially operated for 60 min to verify complete retention of the selected TrOCs by DCMD.

The DCMD module was made up of acrylic glass. Feed and permeate flow channels (145 × 95 × 3 mm) were engraved on each block. Polytetrafluoroethylene (PTFE) membrane was purchased from Ningbo Porous Membrane Technology (Ningbo, China) and was used in this study. The PTFE membrane was hydrophobic in nature, with a surface area, nominal pore size, thickness, and porosity of 221 cm^2^, 0.2 µm, 60 µm, and 80%, respectively.

A bench-scale UV oxidation setup (UVG SLT30 model) purchased from UV Guard (Castle Hill, NSW, Australia) and was integrated with the DCMD setup as shown in Figure 1. It had a working volume of 1.1 L. It comprised an outer 316-grade stainless steel housing protecting an inner quartz reactor. The principal wavelength of the 60 cm long UV lamp (30 W) was 254 nm. According to the supplier, when operated at a flowrate of 16 L/min, this setup provides a UV dose of 40 mJ/cm^2^ (as calculated using UVCalc^®^ software based on a UV transmittance of 85%). With a flowrate of 1 L/min in this study, the estimated UV dose was around 750 mJ/cm^2^. The lamp was placed inside the quartz reactor. It provided continuous exposure to the test solution present inside the outer reactor. All experiments combining DCMD–UV treatment were conducted for a UV exposure time of 60 min.

In this work, the removal of each TrOC in the DCMD–UV system was calculated by establishing a mass balance of TrOC concentration in feed and permeate at the start and end of each run [30]. This is the first study combining DCMD and UV treatment for the removal of TrOCs from their mixture and assessing the effect of inorganic ions (e.g., halide, nitrate, and carbonate) and humic acid on TrOC removal by this process.

### 2.4. TrOC Analysis

The concentrations of TrOCs present in the samples were measured using an HPLC system (Shimadzu, Kyoto, Japan) following a previously published protocol [24]. The limit of quantification for the TrOCs was 10 µg/L. The accuracy of quantification was always confirmed by running standard solutions. Removal of TrOCs was calculated as R (%) = (1 − $\frac{Ct}{C0}$) × 100, where C_0_ and C_t_ are initial mass and mass at time of sampling, respectively.

## 3. Results

### 3.1. Results and Discussion

#### TrOC Removal by DCMD

In DCMD, water passes through the hydrophobic membrane in vapour form. Retention of TrOCs in the feed reactor depends on their volatility and distribution coefficient (log D). TrOCs with pK_H_ value greater than 9 have low volatility and are expected to be well removed by the DCMD system. Log D represents hydrophobicity and can also affect the transport of TrOCs through the MD membrane [17]. 

**Figure 2:**
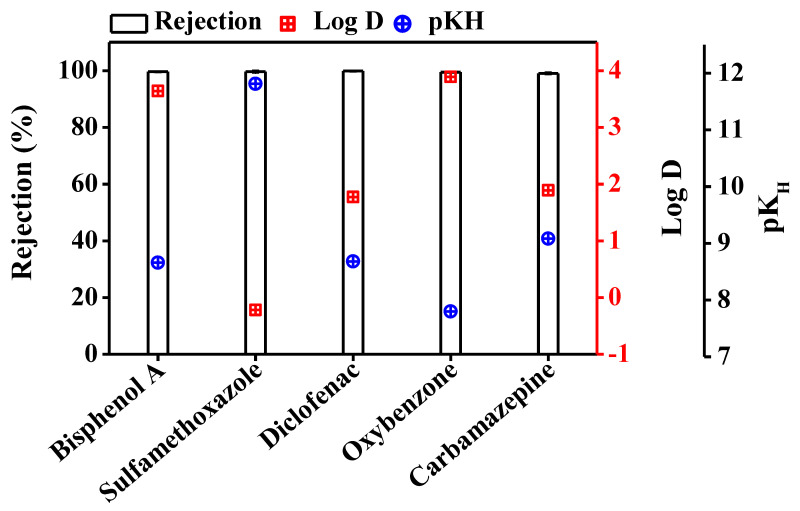
Retention of trace organic contaminants in DCMD system. Retention % was evaluated by establishing a mass balance of trace organic contaminants in the feed at the start and end of the experiment. The DCMD system retained the trace organic contaminants completely. Operating conditions for DCMD system: temperature of the feed and the distillate was set at 30 and 10 °C, respectively; cross flow rate for feed and distillate was maintained at 1 L/min. Error bars represent the standard deviation of duplicate samples.

Interestingly, Figure 2 shows that irrespective of Log D and pK_H_ values, greater than 99% retention of the TrOCs was achieved by the DCMD system. Previously, Wijekoon et al. [17] reported 81% removal for oxybenzone while > 97% rejection for bisphenol A, carbamazepine, and diclofenac at feed and distillate temperatures of 40 and 10 °C, respectively. This difference in TrOC removal can be attributed to the lower feed-side temperature (i.e., 30 vs. 40 °C) used in the current study, which would have reduced the chance of TrOCs escaping in vapor form. 

In our previous study [18], where the DCMD system was run for 18 h (compared to 1 h in the current study), similar TrOC retention was observed with negligible flux decline. Fouling and wetting of membrane can significantly affect TrOC retention when the feed water contains other impurities than TrOC. Future studies are suggested to shed light on this aspect. However, this is beyond the scope of the current study.

### 3.2. Fate of TrOCs in DCMD–UV Photolysis Process

TrOCs retained by the DCMD process eventually accumulate in the feed reactor. This requires additional treatment of the DCMD concentrate before disposal into the environment. Thus, DCMD was combined with UV photolysis to simultaneously retain and degrade TrOCs.

**Figure 3:**
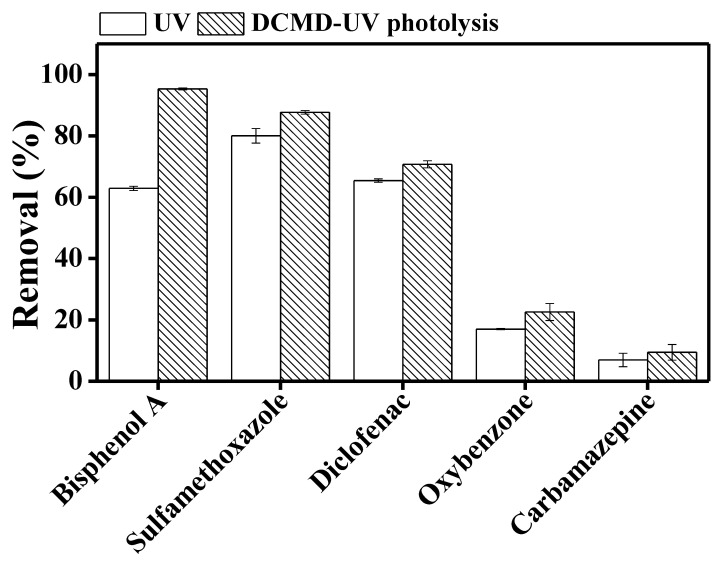
Degradation of trace organic contaminants in UV photolysis and integrated DCMD–UV photolysis. Operating conditions for DCMD: temperature of feed and distillate was set at 30 and 10 °C, respectively; cross flow rate for feed and distillate was maintained at 1 L/min. Operating conditions for photolysis: UV dose was around 750 mJ/cm^2^ and the reaction time was 60 min. Error bars represent the standard deviation of duplicate samples.

The DCMD permeate, i.e., the treated final effluent, was already virtually TrOC-free. The removal efficiency by DCMD–UV discussed in this section refers to the reduction of the concentration of retained TrOCs in feed solution by UV degradation. Samples taken from the feed side revealed substantial degradation of sulfamethoxazole (87%), bisphenol A (95%), and diclofenac (71%) but rather limited degradation of carbamazepine (9%) and oxybenzone (22%) (Figure 3). High removal of sulfamethoxazole, bisphenol A, and diclofenac can be attributed to the presence of more than one photolabile functional group (-CH3, -OH, -COOH, -NH) in their structures, which makes them less stable in the presence of UV irradiation [19]. Low removal of oxybenzone despite the presence of an –OH group in its molecule can be attributed to the presence of fewer photolabile functional groups and a stable benzene ring. Carbamazepine is resistant to photodegradation due to the absence of photosensitive functional groups [31].

Our observation regarding carbamazepine and oxybenzone is consistent with previous studies which reported low removal of these TrOCs by direct UV photolysis at 254 nm [31,32]. Mozia et al. [22] reported 79–96% diclofenac degradation at different initial concentrations of the TrOC, which is consistent with the degradation range that we have observed for this compound. On the other hand, while exploring UV post treatment of DCMD concentrate, Tufail et al. [18] reported lower degradation of bisphenol A compared to the current study. In the current study, the UV system was integrated with the DCMD system. On the other hand, Tufail et al. [18] first operated DCMD independently and then treated the membrane concentrate by UV. Thus, in the current study, the TrOC concentration in the test solution which was exposed to UV was one third. This may be the reason for the better bisphenol A removal.

We compared TrOC degradation in feed solution by UV when the UV system was operated separately versus when DCMD and UV were integrated in the same loop. While for the other TrOCs, UV degradation performance was similar irrespective of the arrangement of the UV and DCMD components, bisphenol A degradation by UV was significantly higher when DCMD and UV were integrated in the same loop (Figure 3). Two previous studies by Mozia et al. [22,33] reported the benefit of integrating the DCMD and UV photolysis in general: the DCMD system retains TrOCs in the feed reactor and increases their concentration, and UV photolysis results in the degradation of the TrOCs; however, they did not discuss the impact of the arrangement of the UV and DCMD components. Nevertheless, noting that in photocatalytic membrane reactors, combining membrane and UV in the same tank results various synergistic advantages [34], it would be interesting to further investigate this aspect. However, this is beyond the scope of the current study.

### 3.3. Effect of Inorganic Ions on the TrOC Removal by DCMD–UV Photolysis

#### 3.3.1. Effect of Nitrate Ion

Upon UV irradiation, nitrate ion produces nitrite and hydroxyl radicals in the reaction system (Equations (1)–(3)) that may help in degrading contaminants.

NO_3_^−^ → [NO_3_^−^]* (1)

[NO_3_^−^]* → NO_2_^−^ + O (2)

[NO_3_^−^]* NO_2_^●^ + O^●−^ → NO_2_ + ^●^OH + OH^−^ (3)

**Figure 4:**
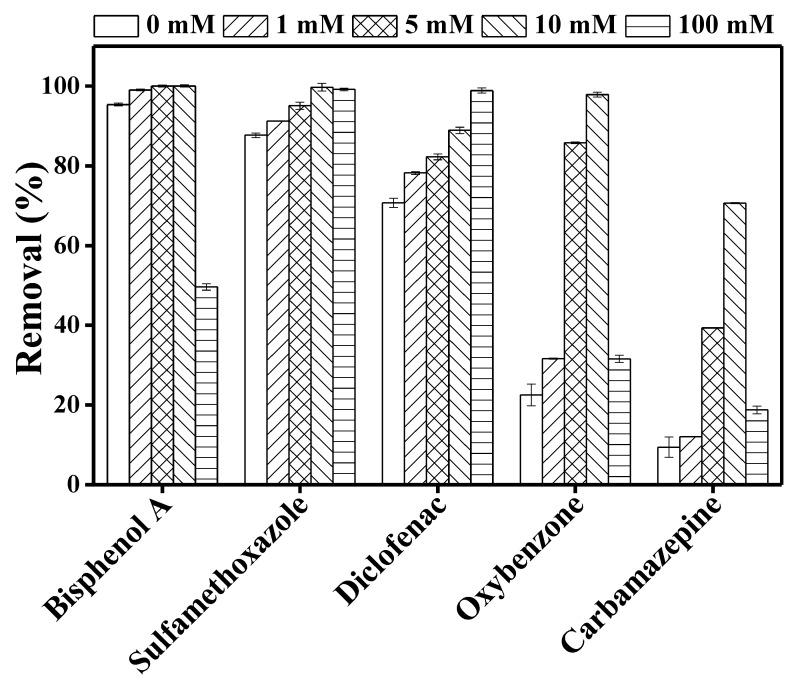
Impact of different concentration (0, 1, 5, 10, 100 mM) of nitrate ions on the degradation of trace organic contaminants in the integrated DCMD–UV photolysis. Permeate flux was 3.6 L/m^2^·h, conductivity was around 4 µS/cm, and TrOC concentration in permeate was below the detection limit. Other operating conditions for integrated DCMD–UV photolysis are given in the caption of Figure 3.

Figure 4 shows a significant increase in TrOC degradation when nitrate ion is added. Furthermore, within the nitrate concentration range of 1–10 mM, TrOC degradation either remained unchanged (bisphenol A, sulfamethoxazole, and diclofenac) or increased gradually (oxybenzone and carbamazepine). The degradation of bisphenol A, oxybenzone, and carbamazepine reduced significantly at a nitrate concentration of 100 mM.

Consistent with our observation regarding sulfamethoxazole, Hao et al. [35] reported a promoting effect of nitrate ion on UV degradation of sulphonamide compounds. Similarly, as shown in Figure 4, removal of diclofenac increased consistently with increasing nitrate ion concentration (0–100 mM). This result is in line with the performance reported by Koumaki et al. [36]. On the other hand, in the current study, bisphenol A degradation reduced by 50% when the nitrate concentration was increased from 10 to 100 mM. Reduced degradation was also observed for carbamazepine and oxybenzone at this level of nitrate concentration (Figure 4). This can be attributed to the “shielding effect” due to the presence of high nitrate ion concentration in the reaction mixture [36]. Nitrate ion also absorbs UV light and, when present in excessive concentrations, this ion can create competition for the available number of photons [37]. The addition of nitrate ion can produce hydroxyl radicals, but these radicals play a small part in the whole degradation process when direct photodegradation plays the key role in TrOC degradation. Thus, TrOC degradation—for example, that of sulfamethoxazole—did not increase proportionately with nitrate ion concentration.

Overall, our results highlight the concentration-specific impact of nitrate ion on UV degradation of TrOCs.

#### 3.3.2. Effect of Halide Ions

Halide ions—namely fluoride, chloride, bromide, and iodide ions—are ubiquitously detected in seawater, surface water, or groundwater over a wide concentration range of 0.1–500 mg/L [25,38]. These ions can undergo photoexcitation by UV radiation having a wavelength below 260 nm. In the presence of UV radiation, halide ions can produce radicals that selectively attack contaminants and degrade them [38]. Redox potential for fluoride, chloride, bromide, and iodide are 2.9, 2.59, 2.04, and 1.37 V, respectively [26,39]. On the other hand, halide ions act as hydroxyl radical scavengers by reacting with hydroxyl radicals to produce halide radicals, which reduces hydroxyl radical-mediated degradation of TrOCs [38,39].

**Figure 5:**
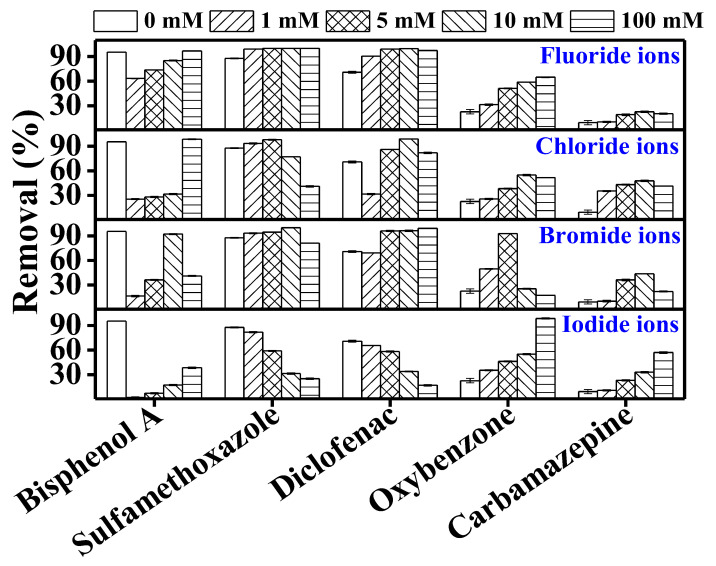
Impact of halide ions (fluoride, chloride, bromide, and iodide) on the degradation of trace organic contaminants in the integrated DCMD–UV photolysis. Permeate flux was 3.7 L/m^2^·h, conductivity was around 4 µS/cm, and TrOC concentration in permeate was below the detection limit. Operating conditions for integrated DCMD–UV photolysis are given in the caption of Figure 3. Halide ion concentrations were 0, 1, 5, 10, 100 mM.

In this study, with a few exceptions, bisphenol A removal significantly decreased in the presence of chloride, bromide, and iodide ions (Figure 5). The reduction in BPA degradation in the presence of halide ions can be attributed to the competition between BPA and the halide ions for UV irradiation. Moreover, the presence of halide ions may generate some other radicals, e.g., HOX, which may inhibit TrOC degradation [40]. For instance, Grebel et al. [41] reported a 90% reduction in the photodegradation of 17β-estradiol in the presence of 0.54 M chloride ion. They attributed this reduction to the ionic strength effect of chloride ions.

Sulfamethoxazole and diclofenac removal was mostly unaffected by the halide ions except for iodide ion. This is in line with previous studies where sulfamethoxazole degradation remained unchanged in the presence of chloride and bromide ions. For example, Li et al. [25] reported that chloride ion (0.54 M) and bromide ion (0.8 mM) did not affect the degradation of sulfapyridine and sulfamethoxazole.

Sulfamethoxazole and diclofenac may involve a triplet-excited state in their photodegradation [25]. Halide ions could quench the triplet-excited state of TrOCs by the formation of complex intermediates between halide ions and the excited state of the TrOC, thus reducing their photodegradation. However, Li et al. [25] suggested that the oxidation potential of sulfamethoxazole at triplet-excited state is not large enough to react with halide ions and form intermediates. Therefore, except iodide, the presence of halide ions did not affect the degradation of sulfamethoxazole. Several studies have demonstrated that the presence of organic matter may create competition for UV light, thus affecting the photodegradation of TrOCs. A stronger light attenuation effect occurs in the presence of organic species with higher absorbance [39,41,42]. Among halide ions, iodide ion shows the highest absorbance that increases with its concentration. Therefore, inhibition of the TrOC degradation can be attributed to the attenuation effect of the halide ions [42].

Except for the significantly reduced removal in the presence of bromide ion beyond a concentration of 5 mM, oxybenzone removal gradually increased with halide concentrations. Oxybenzone can react with chloride ions and generate chlorinated by-products such as chloroform and halogenated methoxyphenols [43]. Therefore, conversion of oxybenzone increased in the presence of chloride ions. Other halide ions also form radicals in the presence of UV irradiation that may react with oxybenzone and promote or inhibit its conversion/degradation [44].

Compared to the other TrOCs, carbamazepine removal in the control experiments (i.e., in the absence of any halides) was originally much lower. Its removal slightly increased in the presence of the halides.

Notably, Li et al. [42] investigated the photodegradation of ibuprofen in the presence of different halide ions. The authors reported that, among all the halides, iodide ion shows the maximum light attenuation effect and can significantly impact the degradation of contaminants. In our study too, overall, with a few exceptions, a major negative impact of iodide ion was observed on the removal of the investigated TrOCs, which can be attributed to its maximum light attenuation effect as discussed above.

#### 3.3.3. Effect of Bicarbonate Ion

Inorganic carbon occurs in water in the form of either carbonate or bicarbonate ions and can affect TrOC photodegradation efficiency [45]. TrOC degradation may reduce because this ion may shield UV radiation [36]. Bicarbonate ion may also scavenge hydroxyl radicals (Equation (4)) and generate carbonate radicals (strong one-electron oxidants): because of this, while the dissipated hydroxyl radical cannot take part in TrOC degradation, the carbonate radicals generated can selectively oxidize TrOCs [26,46].

OH + HCO_3_^−^ → H_2_O + CO_3_^−^ (4)

**Figure 6:**
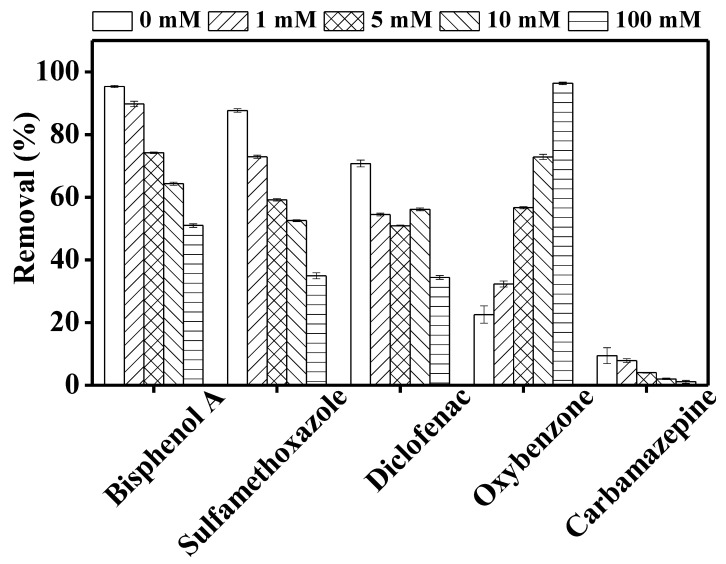
Impact of different concentrations (0, 1, 5, 10, 100 mM) of bicarbonate ions on the degradation of trace organic contaminants in the integrated DCMD–UV photolysis. Permeate flux was 3.5 L/m^2^·h, conductivity was around 4 µS/cm, and TrOC concentration in permeate was below the detection limit. Other operating conditions for integrated DCMD–UV photolysis are given in the caption of Figure 3.

Carbonate radicals react via electron transfer or hydrogen transfer with TrOCs having aromatic amines, thiols, and phenol groups. Only a handful of studies have reported the effect of bicarbonate on the photodegradation of contaminants [26]. In this study, except for oxybenzone, which showed an opposite trend, TrOC removal decreased significantly with the increase in bicarbonate concentration (Figure 6). Our observation is consistent with that of Mozia et al. [33], who reported reduced ibuprofen degradation by MD photocatalysis due to bicarbonate ion. Oxybenzone contains a phenolic functional group and thus its improved photodegradation in our study in the presence of bicarbonate ion can be attributed to the formation of carbonate radicals that degrade phenolic moieties via electron transfer. On the other hand, as in this study, Yang et al. [26] reported a reduction in sulfamethoxazole removal in the presence of bicarbonate ion at a concentration of 50 mM. A similar decreasing trend was observed for the photodegradation of bisphenol A and diclofenac in previous studies [47,48]. It is worth mentioning that in this study, TrOC removal inhibition was not significant at the lowest bicarbonate concentration tested (1 mM). Thus, it is possible that light attenuation at higher bicarbonate concentrations was one of the reasons for the deteriorated photodegradation of the TrOCs [42].

### 3.4. Effect of Humic Acid on TrOC Removal by DCMD–UV Photolysis

Among the natural organic matter in water is humic acid, which has an average molecular weight of 2000–5000 and consists of a large portion of oxygen-containing functional groups. Humic acid is chromophoric in nature; thus, it is excited by UV irradiation having a wavelength ranging between 300 and 500 nm [29,49]. In general, humic acid can promote contaminant degradation through the generation of reactive oxygen species or retard degradation by shielding the UV radiation [39,49]. In the presence of sunlight (which emits predominantly UVA but also UVC), humic acid undergoes excitation and generates various radicals, namely hydroxyl radical (^●^OH), peroxy radical (ROO^●^), and singlet oxygen species (O_2_^●−^) [36]. These radicals can attack TrOCs in the solution and oxidise them. By contrast, humic acid can also attenuate light as it can shield UV radiation. 

**Figure 7:**
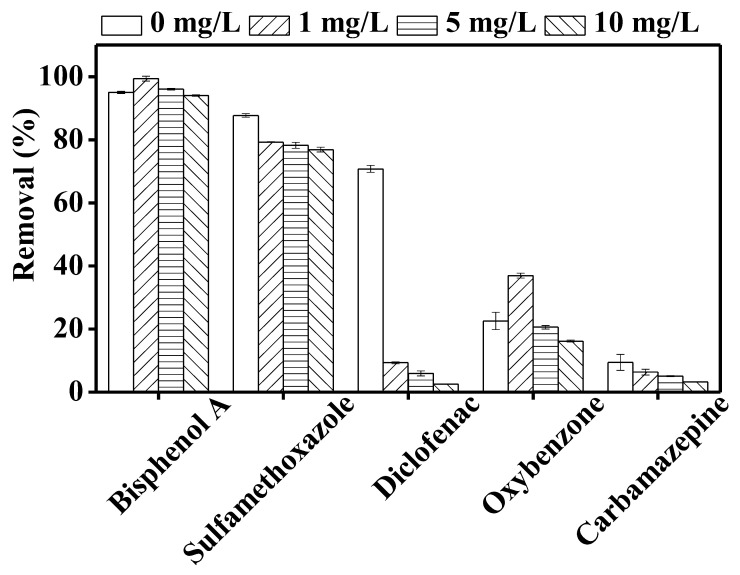
Impact of 1, 5, and 10 mg/L of humic acid on the degradation of trace organic contaminants in the integrated DCMD–UV photolysis. Operating conditions for integrated DCMD–UV photolysis are given in the caption of Figure 3.

Figure 7 illustrates the photodegradation of the investigated TrOCs in the presence of different concentrations (1, 5, 10 mg/L) of humic acid. With the exception of diclofenac, the impact of humic acid on TrOC removal was low. Calza et al. [49] mentioned that the excited triple state of humic acid plays an important role in the degradation of the phenolic contaminants. Both oxybenzone and bisphenol A contain hydroxyl functional groups and thus their degradation was not reduced in the presence of humic acid. On the other hand, in good agreement with our study, Zhang et al. [50] observed a slight inhibitory effect of humic acid (20 mg/L) on the photodegradation of sulfamethoxazole. Similarly, Wang et al. [51] observed reduced degradation of carbamazepine in the presence of humic acid. Consistent with our observation, previously, Koumaki et al. [36] reported that the degradation of diclofenac was greatly reduced in the presence of 20 mg/L of humic acid during 15 h of solar irradiation. Evidently, diclofenac removal is relatively more affected by the light attenuation in the presence of humic acid. In this study, sulfamethoxazole and diclofenac were well removed by direct photolysis but the degradation of diclofenac was greatly affected by humic acid. This difference in degradation can be attributed to their different molar absorption coefficients and pKa values [52,53].

## 4. Conclusions

In this study, we compared the degradation of five trace organic contaminants by UV photolysis and combined DCMD–UV photolysis. Results showed that all five investigated TrOCs were effectively retained (>99%) by the DCMD process, and the retained TrOCs were degraded by UV photolysis with TrOC-specific efficiency. TrOC degradation capacity by the integrated DCMD–UV photolysis process in the presence of humic acid and inorganic ions—namely halide, nitrate, and bicarbonate—was investigated. The nature and extent of the impact of the ions were observed to depend on the type of TrOCs and the concentration of the interfering ions. At a concentration of 1 mg/L, humic acid improved the degradation of the phenolic contaminants (bisphenol A and oxybenzone) while it inhibited the degradation of the non-phenolic contaminants (sulfamethoxazole, carbamazepine, and diclofenac). Conversely, the presence of a high concentration (10 mg/L) of humic acid overall inhibited the degradation of the TrOCs. With an exception, a major negative impact of iodide ion was observed on the removal of the investigated TrOCs. Of particular interest, fluoride and nitrate ions significantly improved TrOC degradation, while bicarbonate ion illustrated a variable influence—from promoting to inhibiting impact—on TrOC degradation.

## Figures and Tables

**Table 1:** Physicochemical properties of the selected trace organic contaminants.

Compound	Molecular Weight (g/mol)	Log D at pH 7	Vapour Pressure (mmHg)	pK_H_ at pH 7
Bisphenol A 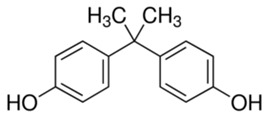	228.29	3.64	5.34 × 10^−7^	8.66
Sulfamethoxazole 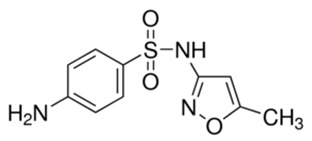	253.28	−0.22	1.52 × 10^−12^	11.81
Diclofenac 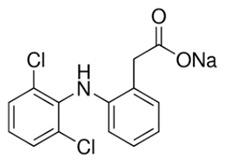	296.15	1.77	1.59 × 10^−7^	8.68
Oxybenzone 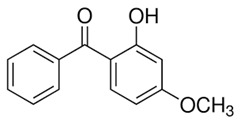	228.24	3.89	5.26 × 10^−6^	7.80
Carbamazepine 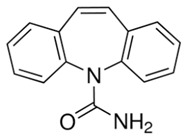	236.27	1.89	5.78 × 10^−7^	9.08

Note: Chemical structure, molecular weight, Log D, vapour pressure, and pKH values were taken from SciFinder Scholar.
